# The promising immune checkpoint LAG-3: from tumor microenvironment to cancer immunotherapy

**DOI:** 10.18632/genesandcancer.180

**Published:** 2018-05

**Authors:** Long Long, Xue Zhang, Fuchun Chen, Qi Pan, Pronnaphat Phiphatwatchara, Yuyang Zeng, Honglei Chen

**Affiliations:** ^1^ Department of Pathology, School of Basic Medical Science, Wuhan University, Wuhan, P. R. China; ^2^ Department of Radiation and Medical Oncology, Zhongnan Hospital, Wuhan University, Wuhan, P. R. China; ^3^ Department of Thoracosurgery, Traditional Chinese Medical Hospital of Wenling, Wenling, Zhejiang, China

**Keywords:** immune checkpoint, lymphocyte activation gene-3, cancer immunotherapy, tumor microenvironment

## Abstract

Cancer immunotherapy and tumor microenvironment have been at the forefront of research over the past decades. Targeting immune checkpoints especially programmed death 1 (PD-1)/programmed death ligand 1 (PD-L1) has made a breakthrough in treating advanced malignancies. However, the low response rate brings a daunting challenge, changing the focus to dig deeply into the tumor microenvironment for alternative therapeutic targets. Strikingly, the inhibitory immune checkpoint lymphocyte activation gene-3 (LAG-3) holds considerable potential. LAG-3 suppresses T cells activation and cytokines secretion, thereby ensuring immune homeostasis. It exerts differential inhibitory impacts on various types of lymphocytes and shows a remarkable synergy with PD-1 to inhibit immune responses. Targeting LAG-3 immunotherapy is moving forward in active clinical trials, and combination immunotherapy of anti-LAG-3 and anti-PD-1 has shown exciting efficacy in fighting PD-1 resistance. Herein, we shed light on the significance of LAG-3 in the tumor microenvironment, highlight its role to regulate different lymphocytes, interplay with other immune checkpoints especially PD-1, and emphasize new advances in LAG-3-targeted immunotherapy.

## INTRODUCTION

Over the past decades, the extraordinary advances in cancer immunotherapy have opened a new era for tumor microenvironment (TME). TME contains tumor cells, cancer-associated fibroblasts (CAFs), endothelial cells, myelomonocytic cells, myeloid-derived suppressor cells (MDSCs), tumor-infiltrating lymphocytes (TILs), extracellular matrix (ECM), and vasculatures [1]. Interactions between various components of the tumor microenvironment are complex and unique, therefore playing a vital role in tumor initiation, malignant progression, metastasis as well as therapeutic efficacy [2, 3].

Immune checkpoints are essential molecules to regulate T cells function in the tumor microenvironment [4]. Immune checkpoint therapy, which blocks inhibitory pathways in T cells to promote anti-tumor immune responses, has remarkably revolutionized cancer treatment paradigms [5]. The fully unraveled primary immune checkpoints are programmed cell death-1 (PD-1)/programmed cell death ligand-1 (PD-L1), and cytotoxic T-lymphocyte antigen-4 (CTLA-4) [5]. Furthermore, PD-1/PD-L1 inhibitors have already been approved by the Food and Drug Administration (FDA) for treating melanoma, non-small cell lung cancer (NSCLC) and other malignancies [6].

**Figure 1:**
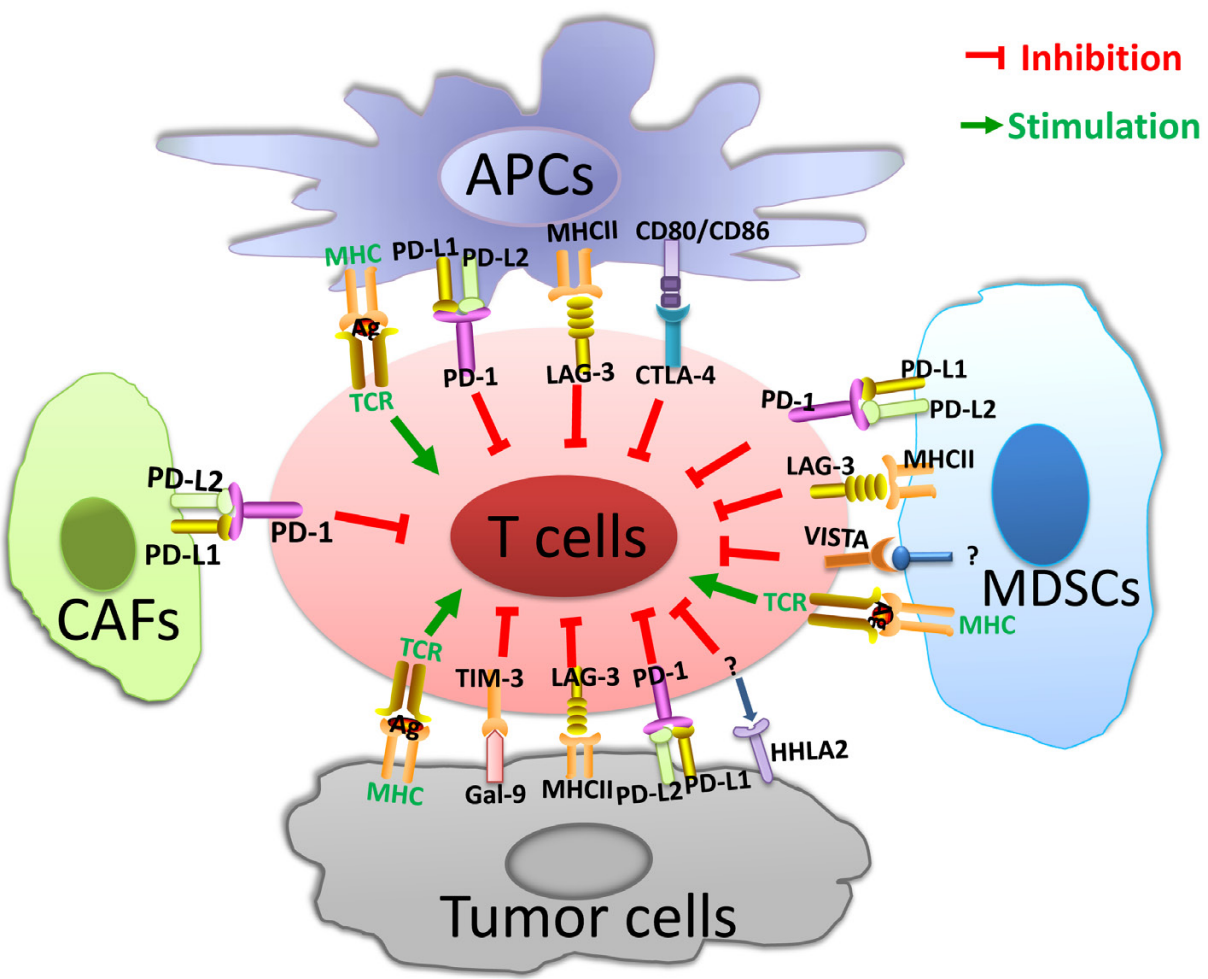
Tumor microenvironment and immune checkpoints In the TME, APCs present tumor antigens to naïve T cells and induce T-cell activation. The MHC and TCR signaling pathway provide the first signal for T-cell activation, while co-inhibitory immune checkpoints collaborate to suppress T-cell activation in the TME. Immune checkpoints are presented on T cells. Ligands are expressed on APCs, tumor cells and other stromal cells, such as CAFs and MDSCs. *Abbreviations:* TME = tumor microenvironment; APCs = antigen presenting cells; MDSCs = myeloid-derived suppressor cells; CAFs = cancer-associated fibroblasts; MHC = major histocompatibility complex; TCR = T-cell receptor; PD-1 = programmed death 1; PD-L1 = programmed cell death ligand-1; PD-L2 = programmed cell death ligand-2; LAG-3 = lymphocyte activation gene-3; CTLA-4 = cytotoxic T-lymphocyte antigen-4; VISTA = V-domain immunoglobulin-containing suppressor of T-cell activation; HHLA2 = human endogenous retrovirus-H long terminal repeat-associating protein 2; TIM-3 = T cell immunoglobulin and mucin domain 3; Gal-9 = Galectin-9; MDSCs = myeloid-derived suppressor cells

Despite the impressive clinical success of immune checkpoint therapy, tumor intrinsic resistance remains a daunting challenge, leading to low response rate in large-scale use of immune checkpoint inhibitors in solid tumors [3, 4]. To permit more patients to benefit from immunotherapy, the focus has changed to targeting alternative novel immune checkpoints in the tumor microenvironment [7], such as lymphocyte activation gene-3 (LAG-3) [8], T cell immunoglobulin and mucin domain 3 (TIM-3) [9], V-domain immunoglobulin-containing suppressor of T-cell activation (VISTA) [10], and human endogenous retrovirus-H long terminal repeat-associating protein 2 (HHLA2) [11](Figure 1).

LAG-3 (CD223) may be a significantly promising immune checkpoint, which is a co-inhibitory receptor to suppress T cells activation and cytokines secretion, thereby ensuring a state of immune homeostasis [12]. LAG-3 exerts differential inhibitory impacts on various types of lymphocytes [13]. Meanwhile, LAG-3 can effectively prevent the onset of autoimmune disorders [14]. The precise molecular mechanisms of LAG-3 signaling and interaction with other immune checkpoints are mostly unclear. However, LAG-3 shows a striking synergy with PD-1 in multiple settings to inhibit immune responses [15]. LAG-3-targeted immunotherapy started in 2006 with a LAG-3 Ig fusion protein (IMP321), and there are currently several LAG-3-modulating treatments in various phases of clinical development [12, 16-18]. In particular, combination therapy of anti-LAG-3 (BMS-986016) plus anti-PD-1 (nivolumab) has shown impressive clinical efficacy in melanoma patients who are resistant to anti-PD-1/PD-L1 therapy [19, 20]. In this review, we provide a detailed description of the significance of the promising immune checkpoint LAG-3 in the tumor microenvironment, discuss its role on different types of lymphocytes and autoimmune disorders, highlight its interplay with other immune checkpoints, as well as outline the new advances targeting LAG-3 in cancer immunotherapy.

## LAG-3 SIGNALING AND ITS EXPRESSION IN HUMAN TUMORS

**Figure 2:**
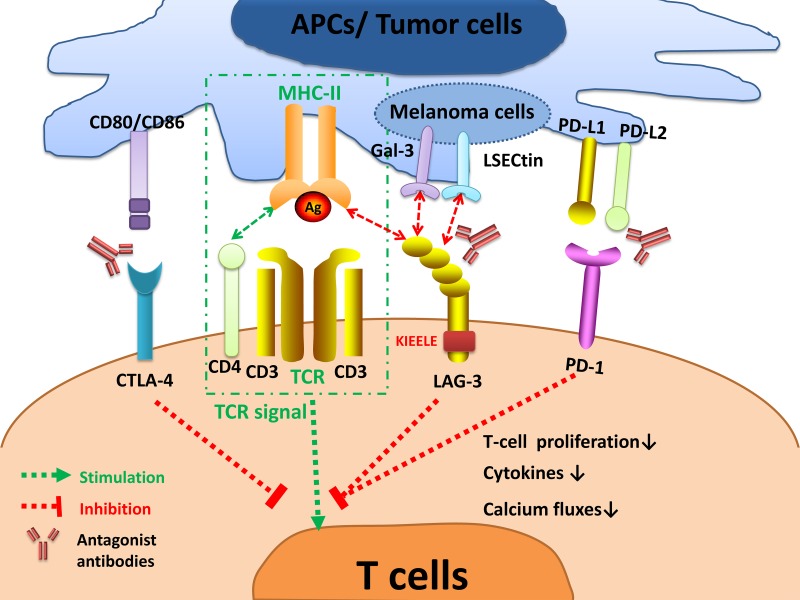
LAG-3 signaling and the interplay with other immune checkpoints The interaction of LAG-3 with MHC-II prohibits the binding of the same MHC molecule to a TCR and CD4, thus suppressing TCR signal. LAG-3 transmits an inhibitory signal via the KIEELE motif in the cytoplasmic tail. Crosslinking of LAG-3 and CD3/TCR complex can impair T cell proliferation, cytokines secretion, and calcium ion fluxes. LAG-3 also interacts with two novel ligands postulated, Gal-3 and LSECtin, expressed on melanoma cells to modulate CD8^+^ T-cell function within the TME. Strikingly, LAG-3 inhibits T-cell activation collectively with other immune checkpoints, especially PD-1. Antagonist antibodies targeting inhibitory immune checkpoints can restore T-cell function and have shown benefits in active clinical trials. *Abbreviations:* APCs = antigen presenting cells; MHC = major histocompatibility complex; TCR = T-cell receptor; PD-1 = programmed death 1; PD-L1 = programmed cell death ligand-1; LAG-3 = lymphocyte activation gene-3; CTLA-4 = cytotoxic T-lymphocyte antigen-4; Gal-3 = Galectin-3; LSECtin = Liver sinusoidal endothelial cell lectin.

LAG-3 is a surface molecule located closely to CD4 but sharing less than 20% homology at the amino acid level [21]. Similar to CD4, LAG-3 binds to major histocompatibility complex-II (MHC-II) on antigen-presenting cells (APCs), but with a much stronger affinity [21]. LAG-3 is expressed on the cell membranes of TILs [22], activated CD4^+^ [23] and CD8^+^ T cells [24] as well as regulatory T cells (Tregs) [25]. It is also expressed on natural killer (NK) cells [26], B cells [27], and dendritic cells (DCs) [28]. LAG-3 belongs to the immunoglobulin superfamily (IgSF) and associates with the CD3/T cell receptor (TCR) complex [29]. LAG-3 interacts with MHC-II to prohibit the binding of the same MHC molecule to TCR and CD4, thus directly hindering TCR signaling in immune response [13]. Crosslinking of LAG-3 and CD3 can impair T cell proliferation and cytokine secretion by inhibiting calcium ion fluxes [29]. The exact signaling transduction mechanism of LAG-3 is still not well elucidated. Nonetheless, the cytoplasmic tail of LAG-3 is quite distinct from other immune checkpoints, suggesting its unique molecular characteristics. It has three conserved domains: the first region may be a possible serine phosphorylation site; the second is KIEELE motif (Figure 2), which is crucial in regulating CD4^+^ T cell function; and the third is glutamic acid-proline (EP) repeat, binding to LAG-3-associated protein (LAP), thereby aid to localizing LAG-3 [21]. LAG-3 intrinsic signaling transmits via the cytoplasmatic KIEELE motif [30]. It prevents T cells to enter the S-phase of the cell cycle and consequently results in suppression of T-cell expansion [30, 31]. However, the intracellular binding partners of KIEELE motif are unidentified.

Besides, the LAG-3/MHC-II interaction may also act as bidirectional inhibitory signaling shared by immune cells and tumor cells [32]. Over-expression of LAG-3 in T cells can protect MHC-II-expressing melanoma cells from drug-induced or FAS-mediated apoptosis through MAPK/ERK and PI3K/AKT survival pathways [32]. Meanwhile, LAG-3-expressing Tregs may utilize an immune tyrosine-based activation motif (ITAM)-modulated suppressive signaling pathway, containing FcγRγ and ERK-modulated recruitment of SHP-1, to indirectly inhibit the function of DCs [33].

Aberrant LAG-3 expression has been found in a broad spectrum of human tumors such as melanoma, NSCLC, colorectal cancer, breast cancer, hepatocellular carcinoma, follicular lymphoma, head and neck squamous cell carcinoma, etc, which is significantly associated with aggressive tumor progression and clinicopathological characteristics (summarized in Table 1) [32, 34–48].

**Table 1:** Expression of LAG-3 in different human tumors and its clinicopathological associations

Type of tumor	LAG-3 Expression	Level	Clinicopathological associations	Ref.
Melanoma	Various TILs	High	Resistance and survival of melanoma cells	[32]
Hodgkin's lymphoma	TILs, PBLs	High	Suppression of EBV-specific T cell function	[34]
Chronic lymphocytic leukemia	CLL cells, TILs, PBLs	High	The unmutated immunoglobulin variable heavy chain leukemic subtype and short time to treatment	[35]
Colorectal cancer	TILs	High	Differentiation, lymph metastasis, invasion, TNM, and Duke stage	[36]
Ovarian cancer	TILs, PBLs	High	Impaired function of NY-ESO-1-specific CD8^+^ T cells	[37]
Hepatocellular carcinoma	TILs	High	HBV-specific CD8^+^ T cells dysfunction	[38]
Renal cell carcinoma	TILs	High	Poor prognostic impact	[39]
Gastric cancer	CD4^+^ and CD8^+^ T cells	High	Positive correlation with PD-1 expression	[40]
Follicular lymphoma	Intratumoral T cells	High	Poor outcome	[41]
Prostate cancer	TILs, PBLs	High	Histological signs of malignancy	[42]
Head and neck squamous cell carcinoma	TILs	High	High pathological grades, lager tumor size, positive lymph node status and poor prognosis	[43]
Non-small cell lung cancer	TILs	High	TIL abundance, PD-1/PD-L1 expression, and reduced survival	[44]
Malignant pleural mesothelioma	TILs	High	PD-1/PD-L1 expression, and immune cell activation	[45]
Breast cancer	TILs	High	ER− subtypes, PD-1/PD-L1^+^ tumors and improved survival	[46]
Anal squamous cellcarcinoma	TILs	High	PD-1/PD-L1 expression	[47]
Pancreatic cancer	TILs	High	PD-1 and CTLA-4 expression	[48]

TIL = Tumor-infiltrating lymphocytes; PBLs = Peripheral blood T cells; EBV = Epstein-Barr virus; HBV = Hepatitis B Virus; CLL = Chronic lymphocytic leukemia; LAG-3 = Lymphocyte activation gene-3; TNM = The TNM Classification of Malignant Tumors; PD-1 = Programmed death 1; ER = Estrogen-receptor; CTLA-4 = Cytotoxic T lymphocyte antigen 4.

## ROLE OF LAG-3 ON TILS

TILs are essential components in the complex landscape of the tumor immune microenvironment, which consists of CD4^+^ T cells, CD8^+^ T cells, Tregs, NK cells, B cells, macrophages and DCs [49]. Over-expression of LAG-3 is detected on various TILs, exhibiting significant immune regulatory impacts (Table 2).

**Table 2:** The correlation between LAG-3 and TILs

Types of TILs	LAG-3 expression	Correlations	Influence factors	Ref.
**CD4^+^ T cells**	Elevated	LAG-3 negatively regulates CD4^+^ T cell activation.	LAG-3/MHC-II interaction, KIEELE motif, gamma-chain cytokines (IL-2, IL-7, IL-12 and IFN-γ), and Tregs	[23, 50–53]
**CD8^+^ T cells**	Elevated	LAG-3 dampens the effector function of CD8^+^ T cells.	Co-inhibitory molecules especially PD-1, LSECtin and galectin-3	[15, 32, 37, 38, 40, 41, 44, 54–60]
**Tregs**	Elevated	LAG-3 promotes Tregs suppressor activity.	Co-inhibitory molecules, cytokines TGF-β and IL-10	[23, 25, 61–64]
**NK cells**	Elevated	LAG-3 inhibits NKT cells proliferation.	IL-12	[26, 65–69]
**pDCs**	Elevated	LAG-3 suppresses pDCs activation.	IL-6 enrichment and IFN-α deficiency	[33–70, 71]

MHC-II = Major histocompatibility complex-II; PD-1 = Programmed death 1; LSECtin=Liver sinusoidal endothelial cell lectin; Tregs = T regulatory cells; NKT = Natural killer cells that can also express T cell receptors; pDCs = plasmacytoid dendritic cells; IL = Interleukin; IFN = Interferon; TGF = Transforming growth factor.

### LAG-3 on CD4^+^ T cells

Tumor-infiltrating CD4^+^ T cells exhibit traits of chronic exhaustion during tumor progression, accompanied by up-regulation of inhibitory receptor LAG-3 [50]. As an MHC-II ligand, LAG-3/MHC-II interaction negatively modulates CD4^+^ T cells expansion and suppresses cytokine response [50, 51]. Increasing evidence has clarified that the specific KIEELE motif in the cytoplasmic tail is indispensable for abrogating effector CD4^+^ T cells [52]. It can mediate intracellular downstream signal transduction, prevent the entry of T cells into the growth phase of the cell cycle, and depletion of this region is ineffective to negatively regulate T cell function *in vitro* or *in vivo* [52]. Moreover, LAG-3 expression on CD4^+^ T cells links to gamma-chain cytokines including interleukin-2,7,12 (IL-2,7,12) and (interferon-γ) IFN-γ [23]. Another study demonstrates that LAG-3 blockade can potentially affect CD4^+^ T-cell populations, lead to a relative skewing from a Treg phenotype and modulate the function of CD4^+^ T cells to be suppressed [23, 53]. Of note, LAG-3 signal can elevate sensitivity to Treg cells, which can mediate chronic exhaustion and inhibition of CD4^+^ T cells during cancer recurrence [50].

### LAG-3 on CD8^+^ T cells

Un-activated CD8^+^ T cells express slight levels of LAG-3, while LAG-3 expression remarkably elevates in response to antigenic stimulus [54]. In self-tolerance models, LAG-3 blockade enhances the effector function of CD8^+^ T cells and elicits higher production of IFN-γ, suggesting the specific role of LAG-3 in limiting self-tolerance. Moreover, the impact is not dependent on CD4^+^ T cells [55]. Accumulating evidence has delineated that LAG-3 is over-expressed on tumor-infiltrating CD8^+^ T cells in various tumor types, such as ovarian cancer, hepatocellular carcinoma, renal cell carcinomas and other solid tumors [32, 37, 38, 40, 41, 44, 56, 57]. Despite the studies which provide insight into the intrinsic mechanism of LAG-3/CD8 interaction remain scarce, it is newly reported that there may exist two additional LAG-3 ligands in the tumor microenvironment to help regulate CD8^+^ T cells-galectin-3 and Liver sinusoidal endothelial cell lectin (LSECtin), which could effectively abolish anti-tumor immunity of CD8^+^ T cells via LAG-3 [58, 59]. Furthermore, it is conspicuous that CD8^+^ T cells can express LAG-3 concurrent with multiple co-inhibitory immune checkpoints especially PD-1 [60], which are manifested to mark the dysfunction of CD8^+^ TILs [15].

### LAG-3 on Tregs

In the TME, Tregs are considered as the culprit to deteriorate anti-tumor immune responses, which can impede cytokines production and enhance suppressor activity [25, 61]. LAG-3 is essential for maximal Treg suppressive function and confers to a regulatory phenotype [25]. More recent studies have described that LAG-3 promotes Treg differentiation, while LAG-3 blockade mitigates Treg induction [23]. Tumor-infiltrating Tregs are featured in the enhancement of accumulation of forkhead box P3 (Foxp3) together with inhibitory molecules such as PD-1, CTLA-4, LAG-3, and TIM-3 during tumor progression [62]. The finding is strongly supported by the research in NSCLC patients, in which LAG-3 expression on tumor-infiltrating Tregs is elevated, compared to peripheral blood and normal adjacent tissues. Besides, tumor-infiltrating Foxp3^+^LAG-3^+^Tregs secrete a higher level of immunosuppressive cytokines IL-10 and transforming growth factor-β (TGF-β) to collectively magnify Tregs activity [63, 64].

### LAG-3 on NK cells

NK cells are predominant defenders in innate immunity against tumors. The studies exploring the underlying mechanism of LAG-3, firstly found on activated NK cells, are still insufficient. Knockout of LAG-3 gene in mice model resulted in decreased natural killer activity [65]. Interestingly, human NK cells are different from mice. In another research, LAG-3 antibody have no specific influence on human natural killing, of which the mechanism needs further investigation [26]. Compared with no impact on NK cytotoxicity, LAG-3 plays a more critical role in NKT cells that can express both NK receptors and T cell receptors. LAG-3 signaling pathway down-regulates the proliferation of NKT cells by arresting S phase in the cell cycle [66]. In addition, LAG-3 has also been reported to uniquely exhaust invariant NKT (iNKT) cells and reduce IFN-γ production in HIV-infected patients [67]. In the TME, a rise in the expression of LAG-3 links to NK cell memory and exhaustion [68]. Besides, recent studies discovered that LAG-3 expression on NK cells is up-regulated in response to IL-12 [68, 69].

### LAG-3 on pDCs

DCs are critical regulators in anti-tumor response with a robust capability to present tumor-specific antigens. According to recent studies, LAG-3 constitutively expresses on plasmacytoid dendritic cells (pDCs) at a higher level than any other DC subset [70]. LAG-3 negatively regulates pDC activation, is implicated in both intrinsic pDC physiology and cell extrinsic interplay with T cells, and can be regarded as a significant functional marker for pDCs [70]. LAG-3 on Treg cells inhibits DC proliferation and maturation through the engagement with MHC-II, which is mediated by an ITAM suppressive signaling pathway [33]. In melanoma patient samples, LAG-3 is highly expressed on tumor-infiltrating pDCs, contributing to directing an immune-suppressive environment. LAG-3/MHC-II signaling can stimulate Toll-like receptor (TLR)-independent activation of pDCs with increased IL-6 and impaired IFN-α production [71].

### Regulation of LAG-3 expression

LAG-3 expression and its strong affinity with MHC-II substantially up-regulates in inflammatory conditions. IL-2, IL-7, and IL-12 could elevate the expression of LAG-3 on human activated T cells, whereas IL-4, IL-6, tumor necrosis factor-α (TNF-α), and TNF-β have no impact [72]. IL-2 controls CD4^+^ T cells frequency and enhances sensitivity to Treg suppression. IL-7 takes part in the progress and maintenance of T cells and is related to a longer-lived memory phenotype [23]. In particular, IL-12 is termedas the most powerful IFN-γ inducer, which can up-regulate LAG-3 expression, raise the frequency of LAG-3 positive T cells and NK cells [53, 69]. What's more, LAG-3 expression is also mediated by the zinc-dependent a disintegrin and metalloproteinases (ADAM) through TCR signaling-dependent mechanisms [73]. LAG-3 is cleaved from the cell surface by the two metalloproteases ADAM 10 and ADAM 17 to allow for sufficient T-cell activation. Subsequently, a soluble LAG-3 (sLAG-3) is generated and may have competence with membrane LAG-3 to colligate with its ligand [74].

## NOVEL LAG-3 LIGANDS EXPRESSED IN THE TUMOR MICROENVIRONMENT

### Galectin-3

The intriguing fact that LAG-3 modulates the proliferation of CD8^+^ T cells without the engagement of MHC-II has given rise to the exploration of additional LAG-3 ligands [12]. Galectin-3 is a galactoside-binding soluble lectin, which serves as a regulator of antigen-specific T-cell activation [75]. Considering LAG-3 can be extensively glycosylated, which is regarded as a proper target to bind with galectin-3. LAG-3 expression correlates with galectin-3, and functional LAG-3 is indispensable for galectin-3-mediated inhibition of cytotoxic T lymphocyte immune response [58]. Galectin-3 is widely expressed in different cell types; thus interaction with LAG-3 serves to broaden LAG-3's immune regulatory impacts on tumor-infiltrating CD8^+^ T cells within the TME [75].

### Liver sinusoidal endothelial cell lectin

LSECtin, which belongs to the C-type lectin receptor superfamily and is highly expressed in the liver as well as melanoma cells, is suggested to be an alternative LAG-3 ligand [59, 76]. LSECtin exerts significant inhibitory function in anti-tumor immunity similar to LAG-3. In melanoma samples, LSECtin is commonly expressed in tumor cells, engages in tumor immune escape and promotes tumor growth. LSECtin colligates with LAG-3 to diminish IFN-γ production from activated T cells [59].

## ROLE OF LAG-3 IN AUTOIMMUNE DISORDERS

Beyond the inhibitory activity of LAG-3 on different types of lymphocytes, LAG-3 may also be necessary to negativelyregulate autoimmunity in many disease-prone environments [14]. For instance, loss of LAG-3 substantially can accelerate type 1 diabetes in Non-Obese Diabetic (NOD) mice with 100% incidence. LAG-3 deficient mice can exhibit increased antigen-reactive CD4^+^ and CD8^+^ T cells infiltration in the islets, accompanied by invasive and rapid insulitis. Meanwhile, LAG-3 blockade also can exaggerate a rapid diabetes onset in wild-type NOD mice [14]. In the B6.SJL mice model, LAG-3 blockade or deficiency may cause elevated susceptibility to Hg-induced autoimmunity, as well as unresponsiveness to tolerance induction [77]. Furthermore, compared with LAG-3 deficiency alone, dual knock out of LAG-3 and PD-1 has been found to quickly induce lethal myocarditis on the BALB/c mice model. This phenotype let us discover synergistic cooperation of LAG-3 and PD-1 in preventing overt autoimmunity and keeping immune homeostasis [78].

## THE INTERPLAY BETWEEN LAG-3 AND OTHER IMMUNE CHECKPOINTS

Interestingly, LAG-3 has remarkable interactions with other immune checkpoints especially PD-1. In T cells co-signaling pathway, TCR binds to MHC to trigger T cells activation, whereas LAG-3 and other inhibitory immune checkpoints lead to cooperative suppressive effects on TCR signaling (Figure 2).

### PD-1/PD-L1

Increasing evidence has elucidated that LAG-3 has remarkable cooperation with the quintessential inhibitory immune checkpoints PD-1/PD-L1, which can conjointly mediate immune homeostasis, abrogate autoimmune disease, and enhance tumor-induced tolerance [15, 78, 79]. PD-1 and LAG-3 have been reported to be extensively co-expressed on CD4^+^, CD8^+^ T cells and particularly tumor-infiltrating T cells [40, 41, 44, 57]. The striking synergy between LAG-3 and PD-1 has been reported in murine melanoma, fibrosarcoma, and colorectal adenocarcinoma models, the combinatorial blockade against LAG-3 and PD-1 effectively eradicate most established tumors, which are largely resistant to single agent treatment. Dual genetic knockout of LAG-3 and PD-1 can delay tumor growth and enable mice to live markedly longer [15]. Likewise, in a murine ovarian cancer model, LAG-3 and PD-1 are synergized to dampen CD8^+^ T cell effector function [80]. In many tumor samples from patients, sustained co-expression of LAG-3 and PD-1 can modulate T cells exhaustion state. For instance, in NY-ESO-1 ovarian cancer samples, LAG-3 and PD-1 collaborate to mark dysfunctional CD8^+^ T cells, both of which attenuate CD8^+^ T cells activation, inhibit cytokines secretion, and take part in immune escape of tumor cells [37]. A recent finding in human NSCLC revealed that over-expression of LAG-3 on TILs significant correlates with PD-1/PD-L1 expression, and the patients with both low expression of LAG-3 and PD-L1 indicate a favorable prognosis [44]. Overall, these valuable preclinical data suggest an apparent synergy between LAG-3 and PD-1, providing the backbone for combinational treatment strategies [81]. Currently, a majority of clinical trials are ongoing to explore the therapeutic benefits of simultaneously targeting LAG-3 and PD-1 (Table 3).

**Table 3:** LAG-3-targeted immunotherapy in clinical trials (Clinical Trials.gov)

Trial number/Ref.	Study population	Interventions	Phase	Status/Outcomes
∙**IMP321 (a soluble LAG-3 Ig)**				
NCT00351949 [16]	Stage IV renal cell carcinoma	IMP321	1	Completed, October 2008 *Induction of effector CD8^+^ T cells in all patients *Reduced tumor growth and better progression-free survival with high doses
NCT00349934 [17]	Metastatic breast carcinoma patients receiving first-line paclitaxel	IMP321	1	Completed, January 2010 *Sustained increase/activation of APCs, NK and CD8^+^ effector/memory cells *50% ORR with IMP321 and paclitaxel compared with 25% ORR with paclitaxel alone
NCT00324623 [18]	Melanoma (skin)	Lymphodepletion, vaccine, IMP321 adjuvant	1	Completed, November 2011 *Induction of more robust and durable cellular antitumor immune responses
NCT03252938	∙ Solid tumors ∙ Peritoneal carcinomatosis	∙ IMP321	1	Recruiting Estimated completion, February 2019
NCT02614833	Hormone receptor-positive metastatic breast cancer	Paclitaxel + IMP321/Placebo	2	Recruiting Estimated completion, December 2020
NCT02676869	Stage IV melanoma Stage III melanoma	IMP321 + Pembrolizumab (Anti-PD-1)	1	Recruiting Estimated completion, December 2018
**BMS-986016 ( Relatlimab, anti-LAG-3 mAb)**				
NCT01968109 (CA224-020) [19, 20, 92]	Advanced solid tumors	Relatlimab (BMS-986016) ± Nivolumab (BMS-936558, Anti-PD-1)	1/2a	Recruiting Estimated completion, October 11, 2019 *The response rates triple in LAG-3 positive melanoma patients (LAG-3 expression ≥1%). *A safety profile similar to nivolumab monotherapy.
NCT02966548 (CA224-034)	Advanced solid tumors	BMS-986016 ± Nivolumab (BMS-936558, Anti-PD-1)	1	Recruiting Estimated completion, July 1, 2020
NCT03470922 (CA224-047)	∙ Previously untreated metastatic or unresectable melanoma	Nivolumab (Anti-PD-1) ± Relatlimab	2/3	Not yet recruiting Estimated completion, March 16, 2022
NCT03459222 (CA224-048)	Advanced malignant tumors	Relatlimab ± Nivolumab (Anti-PD-1) + BMS-986205 (anti-IDO1)/Ipilimumab (Anti-CTLA-4)	1/2	Not yet recruiting Estimated completion, May 16, 2022
NCT02488759	Virus-positive and virus-negative solid tumors	BMS-986016 + Nivolumab (Anti-PD-1)	1/2	Recruiting Estimated completion, December 31, 2019
NCT02060188	Recurrent and metastatic microsatellite high (MSI-H) and non-MSI-H colon cancer	BMS-986016 + Nivolumab (Anti-PD-1)	2	Recruiting Estimated completion, December 31, 2019
NCT02061761	∙ Hematologic neoplasms	BMS-986016 ± Nivolumab (BMS-936558, Anti-PD-1)	1/2a	Recruiting Estimated completion, January 15, 2020
NCT02658981	Glioblastoma Gliosarcoma Recurrent brain neoplasm	A1: BMS 986016 A2: Urelumab (Anti-CD137) B1: BMS-986016 + Nivolumab (Anti-PD-1) B2: Urelumab (Anti-CD137) + Nivolumab (Anti-PD-1)	1	Recruiting Estimated completion, December 2019
NCT02935634	Advanced gastric cancer	BMS-986016 + Nivolumab (Anti-PD-1)	2	Recruiting Estimated completion, November 18, 2021
NCT02750514	Advanced non-small cell lung cancer	Nivolumab (Anti-PD-1) ± BMS-986016	2	Recruiting Estimated completion, April 29, 2021
NCT02996110	Advanced renal cell carcinoma	Nivolumab (Anti-PD-1) + Relatlimab/BMS-986205 (anti-IDO1)/Ipilimumab (Anti-CTLA-4)	2	Recruiting Estimated completion, January 18, 2022
NCT03335540	Advanced cancer	Nivolumab (Anti-PD-1) + Relatlimab/Radiation Therapy	1	Recruiting Estimated completion, January 31, 2022
**LAG525 (anti-LAG-3 mAb)**				
NCT03365791	Advanced solid and hematologic malignancies.	LAG525 + PDR001 (anti-PD-1)	2	Recruiting Estimated completion, February 1, 2021
NCT02460224	Advanced solid tumors	LAG525 + PDR001 (anti-PD-1)	1/2	Recruiting Estimated completion, April 23, 2019
**REGN3767 (anti-LAG-3 mAb)**				
NCT03005782	Malignancies	REGN3767 ± REGN2810 (Anti-PD-1)	1	Recruiting Estimated completion: October 6, 2020
**TSR-033 (anti-LAG-3 mAb)**				
NCT03250832	Advanced solid tumors	TSR-033 ± Anti-PD-1	1	Recruiting Estimated completion, May 2021
**MGD013 (a PD-1/LAG-3 bispecific DART® protein)**				
NCT03219268	Advanced solid tumors Hematologic neoplasms	MGD013	1	Recruiting Estimated completion, August 2022
**FS118 (a LAG-3/PD-L1 bispecific antibody)**				
NCT03440437	Advanced malignancies that have progressed on or after prior PD-1/PD-L1 containing therapy	FS118	1	Not yet recruiting Estimated completion, May 16, 2020

PD-1 = Programmed death 1; PD-L1 = Programmed death ligand 1; CTLA-4 = Cytotoxic T lymphocyte antigen 4; IDO-1 = Indoleamine 2,3-dioxygenase; mAb = monoclonal antibody; DART = Dual-Affinity Re-Targeting.

### CTLA-4

LAG-3 regulates anti-tumor immune responses interestingly parallels to CTLA-4, a well-known cancer immune checkpoint. Both CTLA-4 and LAG-3 can inhibit TCR signaling pathway, arrest cell cycle progression, negatively modulate T cell homeostasis, trigger the immunosuppressive function of Tregs, and exert essential effects on DCs [82]. However, LAG-3 might be more significant to suppress the activation of primary T cells and expansion of memory T cells [30]. The existing intersections of their signal transduction pathways may lead to the intriguing functional similarity of LAG-3 and CTLA-4 [83]. They can both participate in immune tolerance through the co-inhibitory signaling pathway. For instance, in anterior chamber-associated immune deviation (ACAID) mice models, LAG-3 and CTLA-4 on CD4^+^CD25^+^Foxp3^+^Tregs cells are remarkably upregulated, which induces the development of ACAID [84]. Besides, pDCs induced-CD8^+^Foxp3^+^Treg cells could co-express LAG-3 and CTLA-4, suppressing alloreactive T cells through a CTLA-4-dependent mechanism [85]. In acute graft-*versus*-host disease (GVHD), both human and murine experimental evidence demonstrates that co-blockade using tetravalent CTLA-4-Ig and LAG-3-Ig could synergistically suppress T cell responses, prevent acute GVHD, and decrease GVHD fatality rates as well [86]. A recent study, which assesses the therapeutic effects of the CTLA-4 antibody ipilimumab, found ipilimumab might increase frequencies of tumor-infiltrating T cells expressing LAG-3 in metastatic melanoma patients [87]. Also, it is notable that a new phase I/II clinical trial (NCT03459222) has recently been opened to investigate the efficacy of triple targeting LAG-3, PD-1, and CTLA-4, which may be a novel combinatorial strategy in cancer treatment and autoimmune disorders.

### Targeting LAG-3 in cancer immunotherapy

Immune checkpoints therapy has been reshaping the intriguing landscape of cancer immunotherapy; however, tumor intrinsic resistance is a challenging problem. The unmet need is to identify more alternative therapeutic targets, therefore unleash the full armamentarium of immune checkpoints therapy. Strikingly, the emerging immune checkpoint LAG-3 is considered as a highly promising therapeutic target [8]. Anti-LAG-3 antibody can not only promote effector T cells activity but also inhibit Treg-induced suppressive function in the TME[88]. What's more, in light of the interaction between LAG-3 and other immune checkpoints, targeting LAG-3 along with other checkpoints especially PD-1 holds considerable promise in cancer immunotherapy [89]. There are some approaches involving LAG-3-targeted immunotherapy in different phases of clinical development (Table 3; Clinical Trials.gov).

The first is IMP321, a soluble LAG-3Ig fusion protein which has been investigated in clinical trials since 2006 [16-18, 90]. IMP321 has already completed three clinical trials in renal cell carcinoma, metastatic breast carcinoma, and melanoma with moderate success [16-18] and is still progressing in new clinical trials to further exploring therapeutic benefits. IMP321 has been found to be a systemic APC activator to enhance the proliferation of DCs, lessen Treg cells immunosuppressive effects, and allow for optimal cross antigen presenting to CD8^+^ T cells [90]. The second is antagonistic LAG-3 antibodies which release the brakes of the anti-tumor immune response, such as BMS-986016, LAG525, REGN3767and TSR-033. Considerable clinical trials are proceeding to evaluate LAG-3 antibody alone or in combination with PD-1 antibody [91] (Table 3). The third is first-in-class bispecific protein binding PD-1 and LAG-3 such as MGD013 and FS118, which are currently undergoing phase I clinical trials.

As the first anti-LAG-3 antibody to develop in 2013, BMS-986016 is actively being evaluated in twelve phase I or II clinical trials in many hematological and solid tumors (Table 3; Clinical Trias.gov). Notably, the combination of BMS-986016 and nivolumab (anti-PD-1) exhibited exciting preliminary efficacy in melanoma patients who were refractory to the previous anti–PD-1/PD-L1 therapy (NCT01968109). The combination therapy showed a safety profile analogous to nivolumab monotherapy, with uncommon grade 3/4 adverse events [19]. Based on the updated data at the 2017 ESMO Congress, the combination therapy can increase objective response rates from 5% to 18% in patients with LAG-3–positive tumors. Patients with LAG-3 expression ≥1% are more likely to derive enhanced clinical efficacy [20]. These results are of particular interest for the ongoing more extensive exploration of LAG-3 as an alternative immunotherapy target and potential predictive biomarker [92]. Moreover, we are looking forward to more well-controlled phase III clinical trials to confirm the clinical benefits of LAG-3-targeted immunotherapy.

## CONCLUSION AND PERSPECTIVES

Cancer immunotherapy and tumor microenvironment have been at the forefront of cancer research over the past several decades. Waves of immune checkpoints therapy especially targeting PD-1/PD-L1 have led to remarkable success in treating advanced malignancies. However, most patients do not respond and even develop resistance, bringing about a daunting challenge. Therefore, the research focus has altered to dig deeply into the TME for additional therapeutic targets that can lead to combination therapy. Conspicuously, LAG-3 plays an exceptional inhibitory role in maintaining immune homeostasis, which is expressed on various types of lymphocytes, accompanied by cooperation with other immune checkpoints especially PD-1. LAG-3 may be a promising therapeutic target in cancer immunotherapy, and the combination of BMS-986016 (anti-LAG-3) plus nivolumab (anti-PD-1) exhibited compelling clinical benefits in melanoma patients who are unresponsive to prior anti–PD-1/PD-L1 therapy with more well-designed clinical trials moving forward.

Since the current research is just a tip of the iceberg, there remain vital questions before optimizing the significance of LAG-3 in the TME and cancer immunotherapy. First, the precise molecular mechanisms by which LAG-3 mediates the TCR signaling pathway and function are largely unknown. Considering the unique intracellular cytoplasmic domain of LAG-3 such as KEELE, we may propose that LAG-3 biology is unusual and distinct from other immune checkpoints. A thorough understanding the mode of LAG-3's action is a top priority and can provide a consolidating foundation for future therapeutic development. Second, due to the immune regulatory role of LAG-3 on various types of lymphocytes, how to best apply the differential function in clinical immunotherapy remains an interesting question. Compared with the effects of LAG-3 on effector T cells and Tregs, the specialized roles of LAG-3 in pDCs and NK cells are relatively unexplored. Third, from a mechanistic view, what leads to the complicated interaction of LAG-3 and other immune checkpoints particularly PD-1? How does LAG-3 influence tumor progression when anti-PD-1 does not work? So can LAG-3 expression serve as a robust predictive biomarker to predict response to anti-PD-1/PD-L1 immunotherapy? Further elucidation of these crucial questions may help to design effective combinatorial therapy strategies and overcome possible resistance. The heightened interests will be on the therapeutic benefits of combinatorial immunotherapy of PD-1 and LAG-3. Last but not least, identifying ideal combinatorial strategies between LAG-3-targeted immunotherapy and other front-line modalities, including chemotherapy, radiotherapy as well as targeted therapy may also show tantalizing promises in clinical practice. Taken together, the significant role of LAG-3 in the TME has paved the way for LAG-3-targeted immunotherapy. More prospective clinical studies targeting LAG-3 alone or in combination with PD-1 are further needed, to facilitate the translation of our understanding from bench to bedside.
